# West Virginia Medical Society

**Published:** 1873-01

**Authors:** 


### *West Virginia State Medical Society.*

This Society held its last annual session in the city of Wheeling. Dr. R. H. Cummins, of Wheeling, was elected President; Dr. Roemer, of Charleston, First Vice-President; Dr. Davis, Second Vice-President; Dr. Moore, Third Vice-President; Dr. Wm. Dent, Secretary, and Dr. J. C. Hupp, Treasurer. Dr. J. M. Lazzell was the retiring President.

Many valuable papers were read. Among them we notice the report of a case, by Dr. S. L. Jepson, of a dropsical woman, in which a *post mortem*, one hour after death, revealed the presence, in the right ventricle and pulmonary artery, of a firm, elastic clot over eight inches long.

The Society is harmonious, in fine working order, and promises much for the physicians of West Virginia.

